# Supplementary material for: Genome-wide profiling of transcribed enhancers during macrophage activation
Source: Epigenetics Chromatin. 2017 Oct 23;10:50. doi: 10.1186/s13072-017-0158-9 (PMC5654053; doi:10.1186/s13072-017-0158-9)
Supplement: Supplementary file 3 — Additional file 3: Figure S1. Comparison of 222,870 TAD-based E–P pairs to a subset of 64,891 correlation-based E–P pairs. Figure S2. 1844 macrophage-specific and 8923 non-macrophage-specific genes. Figure S3. Expression of macrophage-specific and non-macrophage-specific genes associated with different number of enhancers. Figure S4. KEGG pathway maps significantly enriched for G1 and G2 genes. Figure S5. Overlaps of M(IFN-γ)- and M(IL-4/IL-13)-responsive and macrophage-specific genes and enhancers. Figure S6. M(IFN-γ) marker enhancer associated with Cxcl9, Cxcl10, and Cxcl11 M(IFN-γ) marker genes. Figure S7. Time-course expression of Arg1 and associated M(IL-4/IL-13)-specific enhancer. Figure S8. Igf1 marker gene. Figure S9. M(IL-4/IL-13) marker enhancer associated with Igf1 M(IL-4/IL-13) marker gene. Figure S10. Macrophage-specific enhancer, associated with Spi1 gene. [file 13072_2017_158_MOESM3_ESM.pdf]

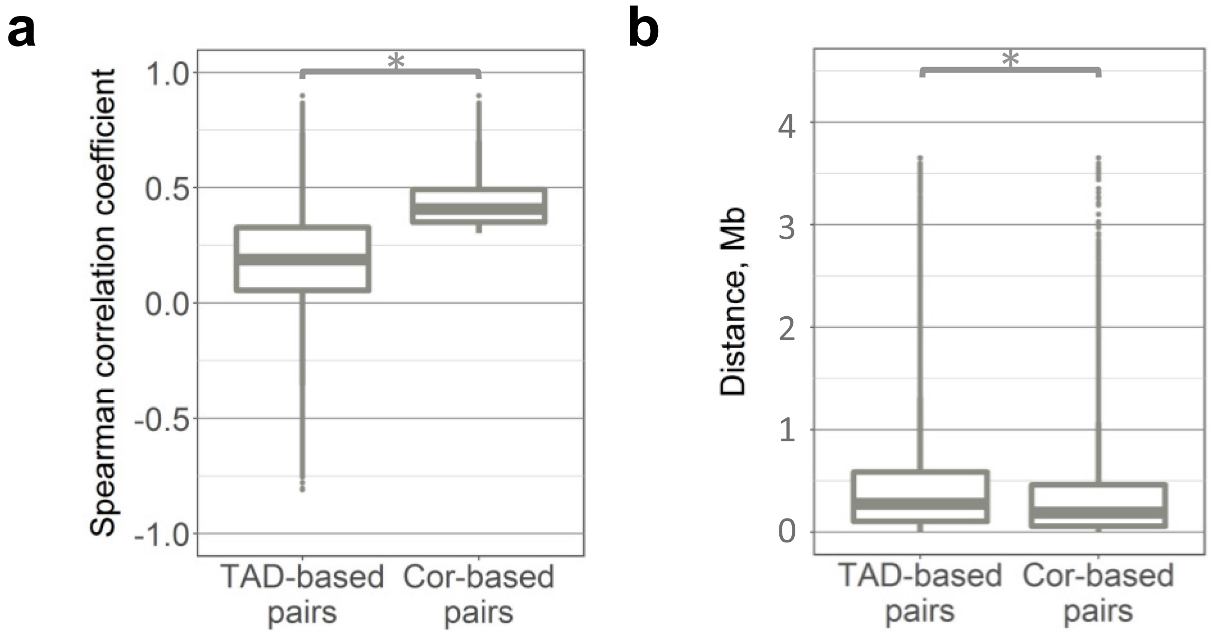

Figure S1. Comparison of 222,870 TAD-based E-P pairs to a subset of 64,891 correlation-based E-P pairs. a Correlation of expression of enhancer eRNAs and promoters. **b** Distance between middle points of paired enhancers and promoters. Asterisks denote Wilcoxon rank sum test p-value $< 2.2*10^{-16}$.

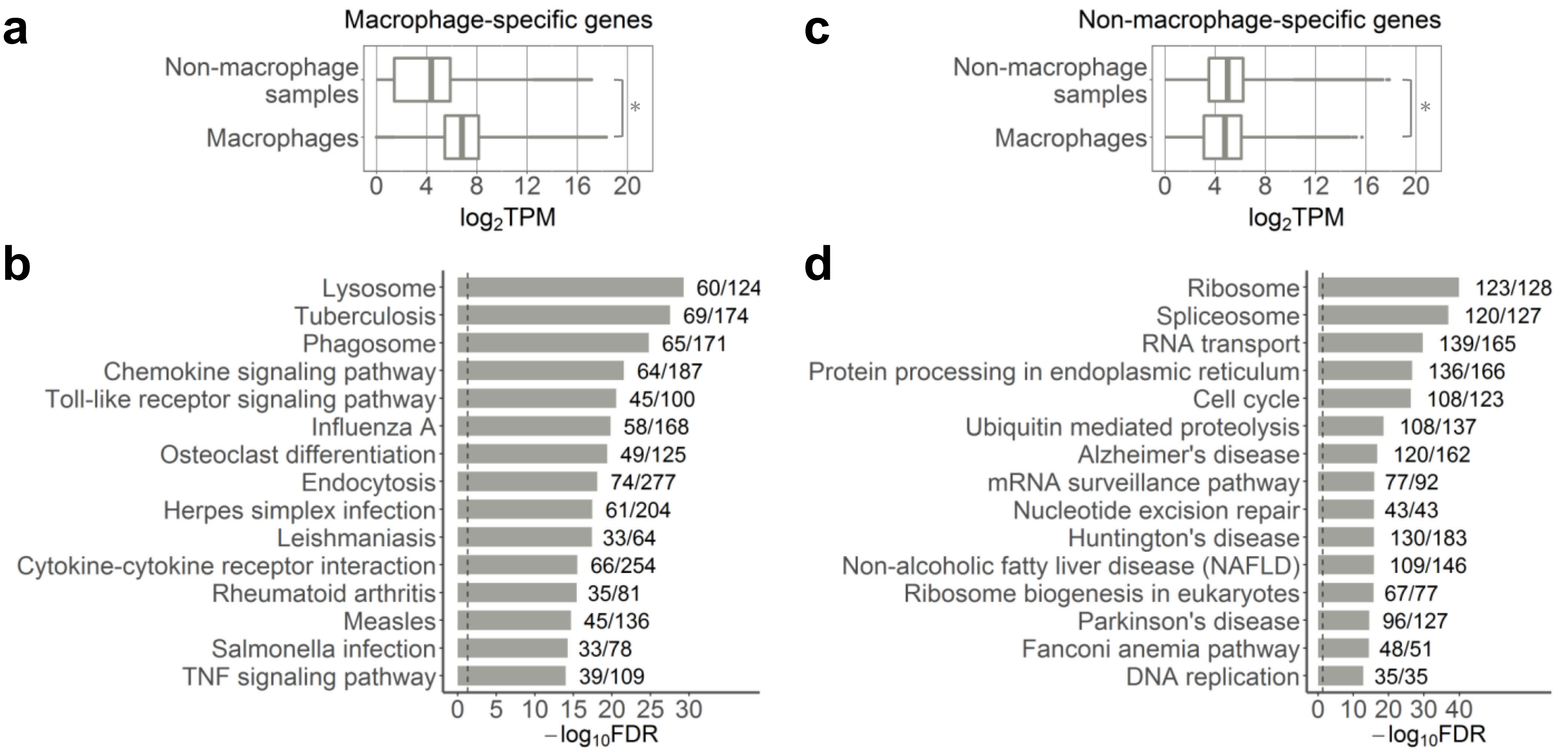

Figure S2. 1,844 macrophage-specific and 8,923 non-macrophage-specific genes. a Expression of macrophage-specific genes. **b** KEGG pathway maps significantly enriched for macrophage-specific genes. **c** Expression of non-macrophage-specific genes. **d** KEGG pathway maps significantly enriched for non-macrophage-specific genes. In **a** and **c** boxplots show expression in 184 macrophage and 744 non-macrophage samples, asterisks denote significant difference in expression (Wilcoxon signed-rank test p-value < $2.2*10^{-16}$). In **b** and **d** top 15 KEGG terms with the lowest FDR are shown, next to the bars are the number of genes in the KEGG pathway covered by our gene list.

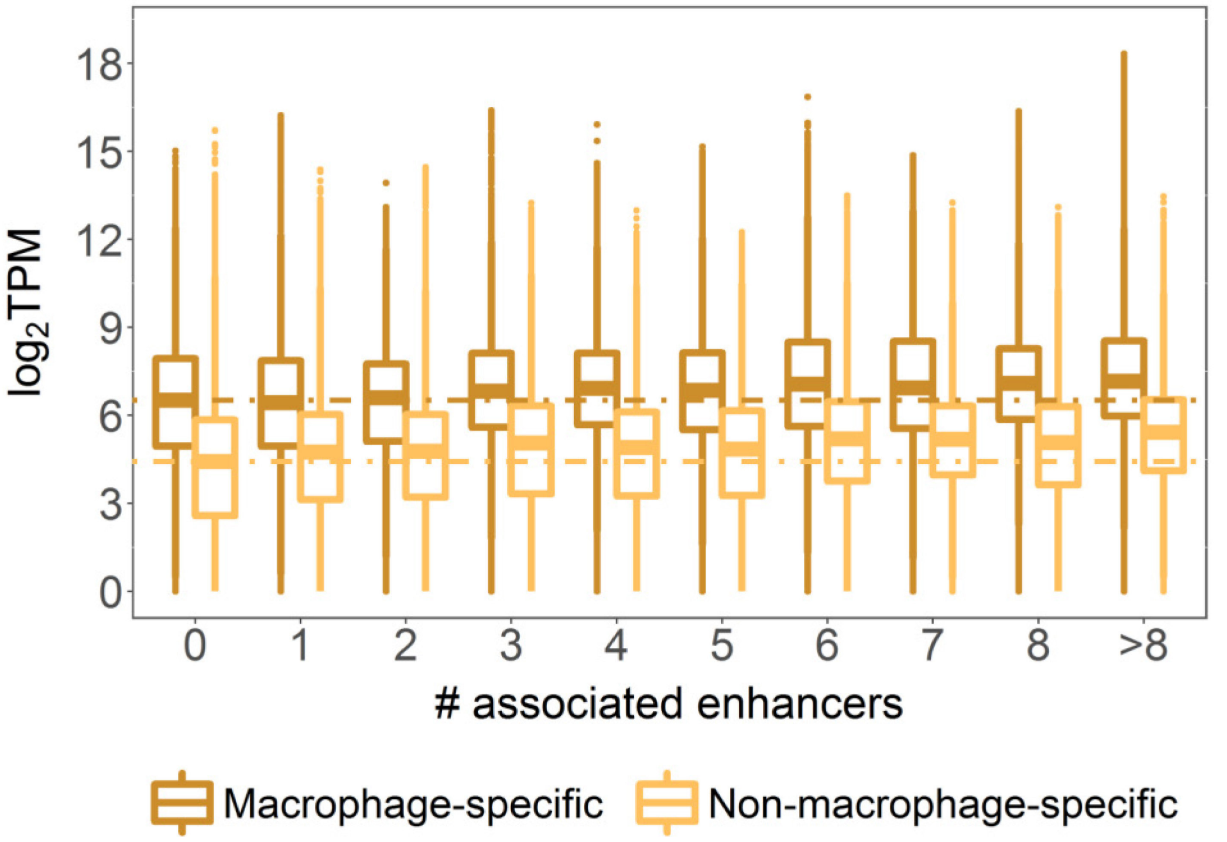

Figure S3. Expression of macrophage-specific and non-macrophage-specific genes associated to different number of enhancers. Dashed lines show median expression of genes not associated to any enhancer.

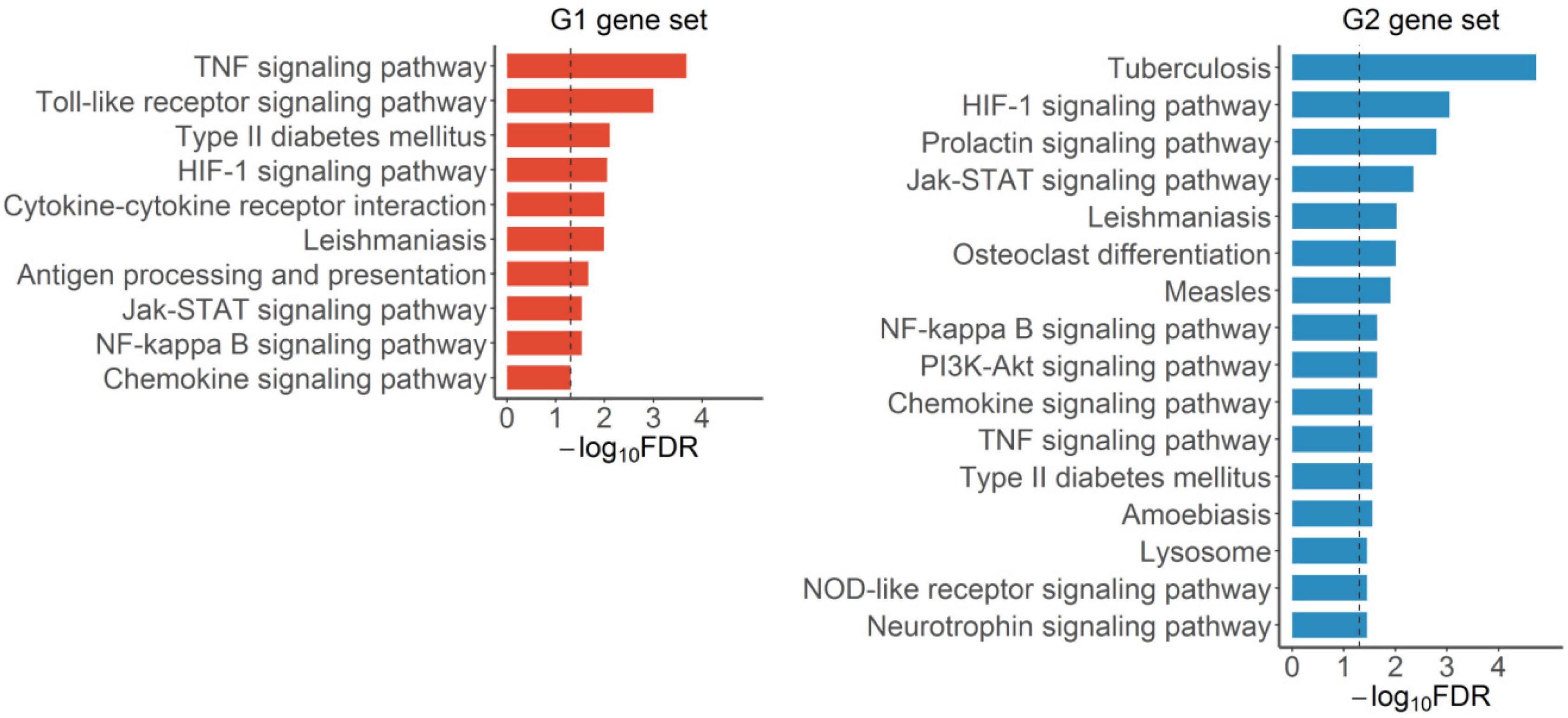

Figure S4. KEGG pathway maps significantly enriched for G1 and G2 genes. Dashed lines indicate FDR = 0.05, used as a threshold.

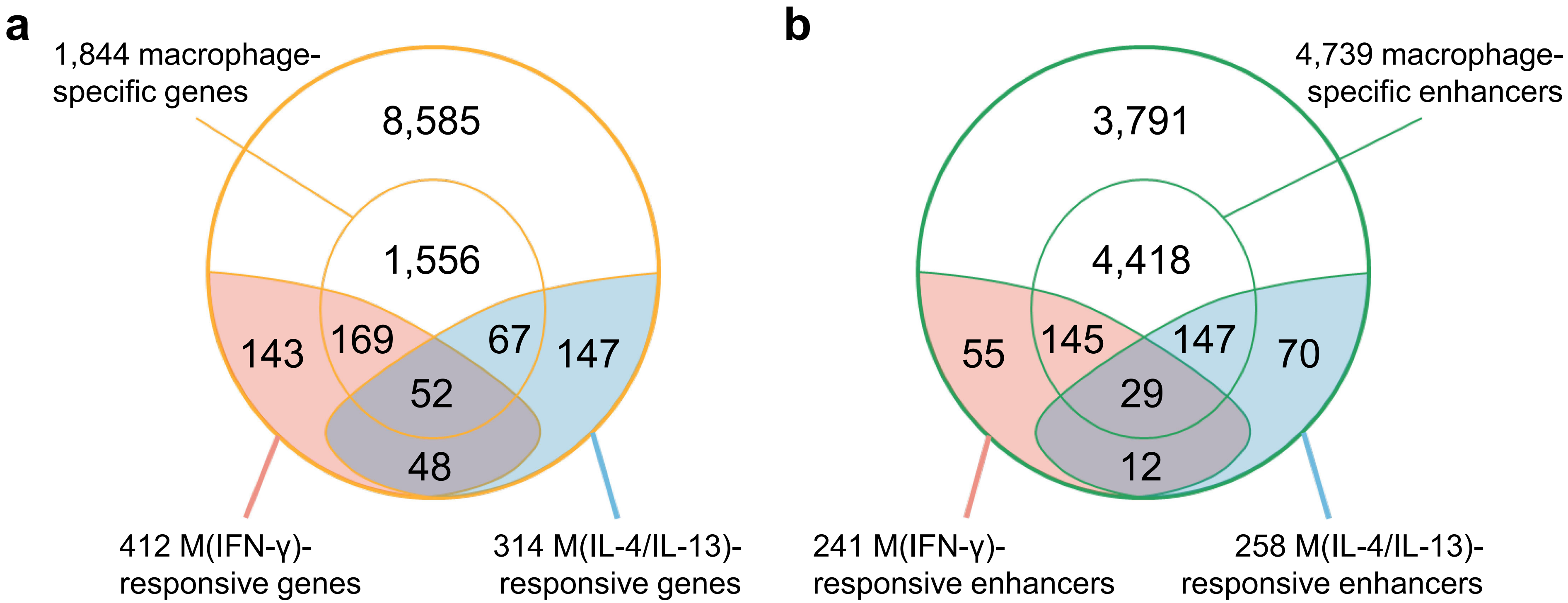

Figure S5. Overlaps of M(IFN-γ)- and M(IL-4/IL-13)-responsive and macrophage-specific genes and enhancers. a Genes, the large circle includes all 10,767 genes considered in this study. **b** Enhancers, the large circle covers all 8,667 enhancers in our BMDM interactome.

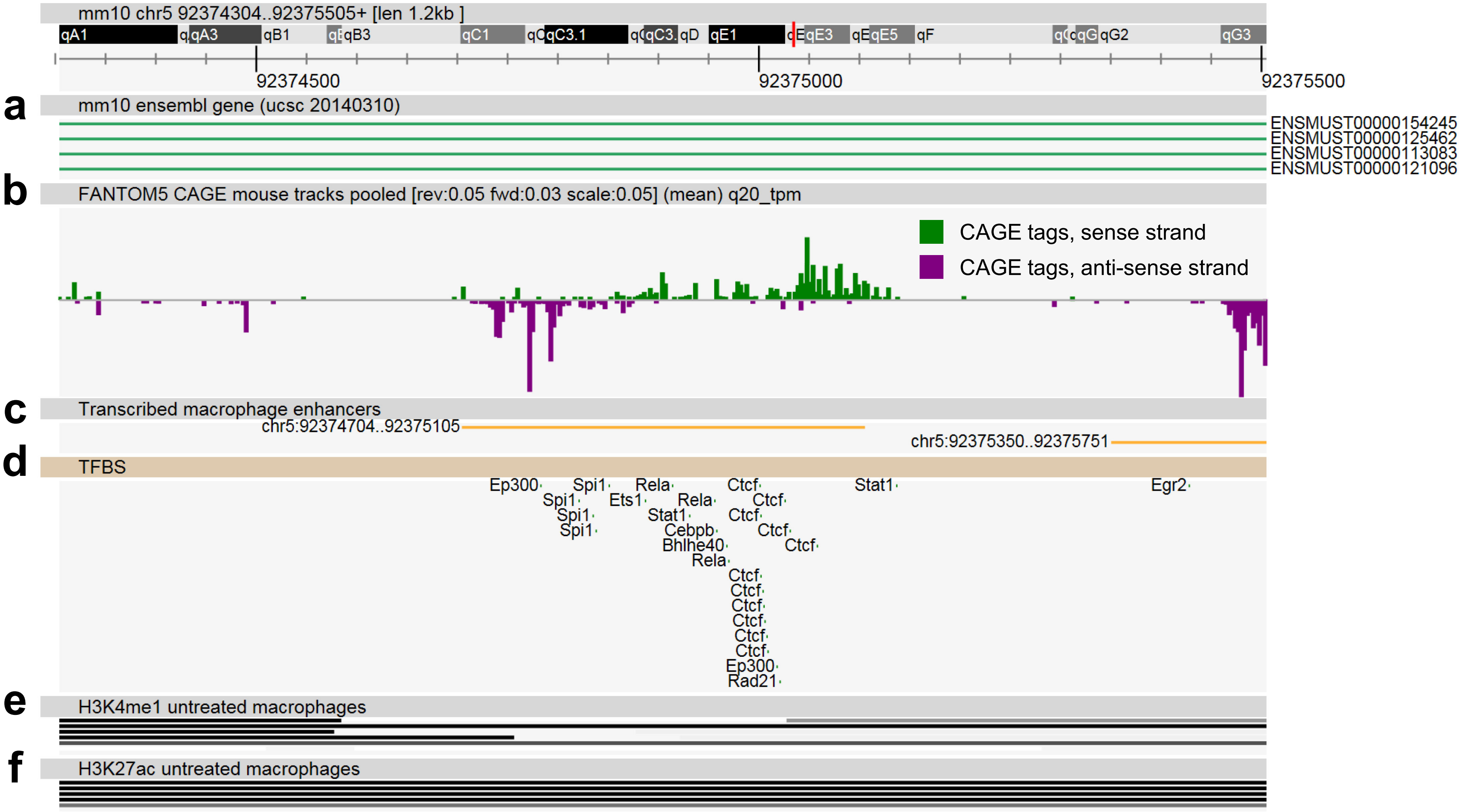

Figure S6. M(IFN-γ) marker enhancer associated to Cxcl9, Cxcl10, and Cxcl11 M(IFN-γ) marker genes. ZENBU genome browser view shows the enhancer and 400bp flanking regions. The enhancer has non-macrophage-specific eRNA expression; expression for eRNA and associated genes is shown in Fig. 4a-b. **a** The enhancer is located in an intron of the Art3 gene. **b** CAGE signal in 184 macrophage samples, split by strand. **c** Enhancer region. **d** Summits of transcription factor binding sites; based on ChIP-seq data that were used for TFBS over-representation analysis, see Methods. **e, f** Significant ChIP-seq peaks for enhancer-specific chromatin marks that were used to define ChIP-seq-based enhancers, see Methods.

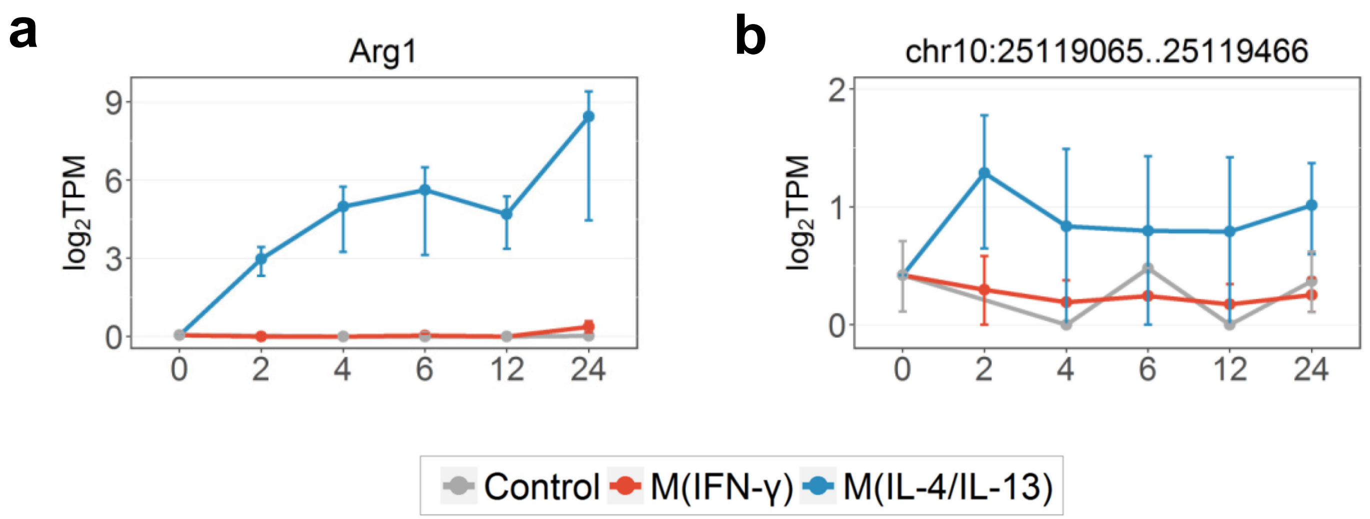

Figure S7. Time-course expression of Arg1 and associated M(IL-4/IL-13)-specific enhancer. Expression data were averaged over replicates and log-transformed. Error bars are the SEM.

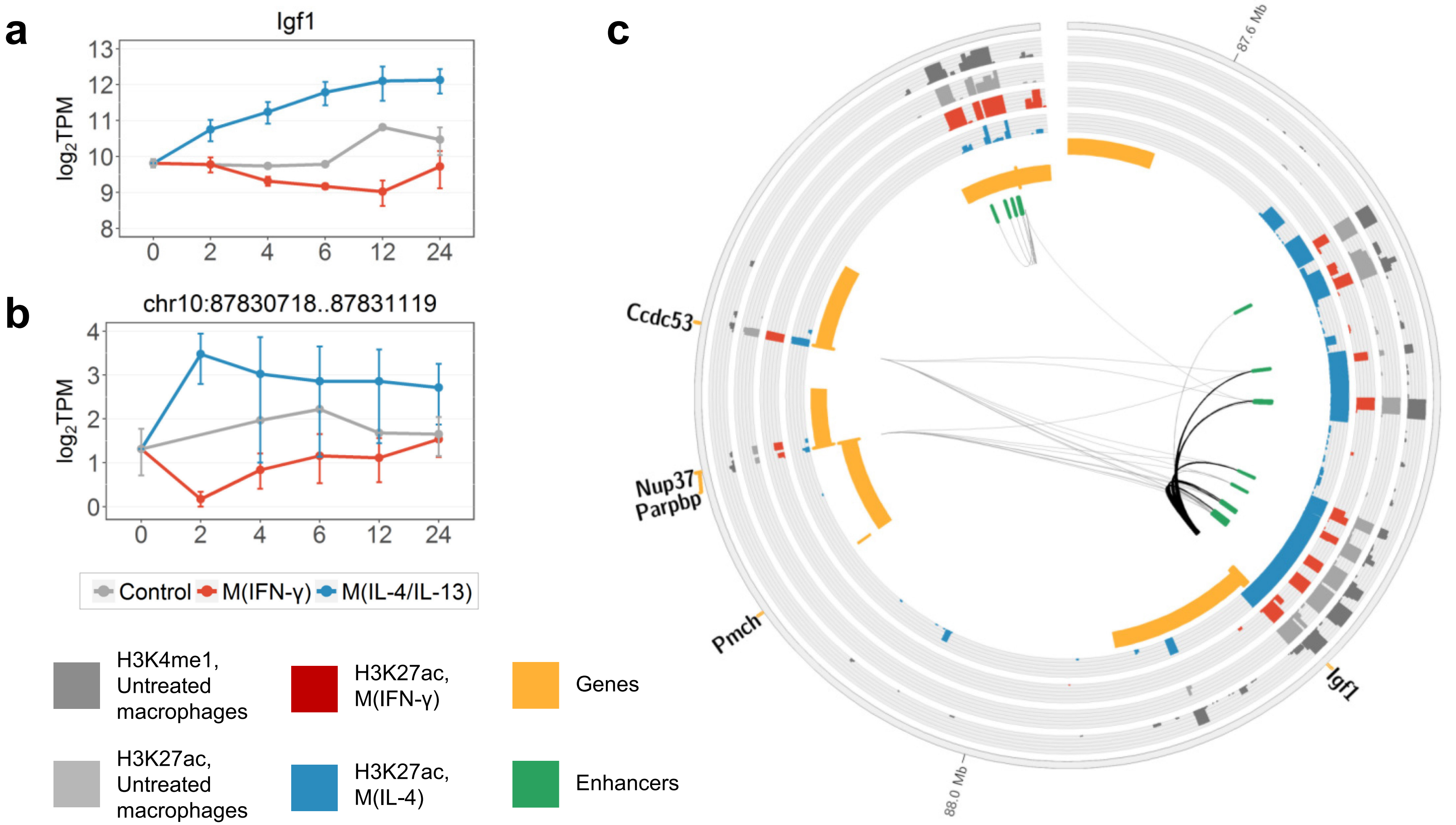

Figure S8. Igf1 marker gene. **a** Time-course expression of Igf1. **b** Time-course eRNA expression of Igf1-associated M(IL-4/IL-13) marker enhancer with the highest expression at 2 hr. In **a** and **b** data were averaged over replicates and log-transformed. Error bars are the SEM. **c** TAD containing Igf1 marker and associated enhancers. Black links connect Igf1 to the 6 M(IL-4/IL-13) marker enhancers. Grey links denote other enhancer-gene interactions identified in macrophages. Genes are split into two tracks based on the strand. Wide orange marks denote gene promoters.

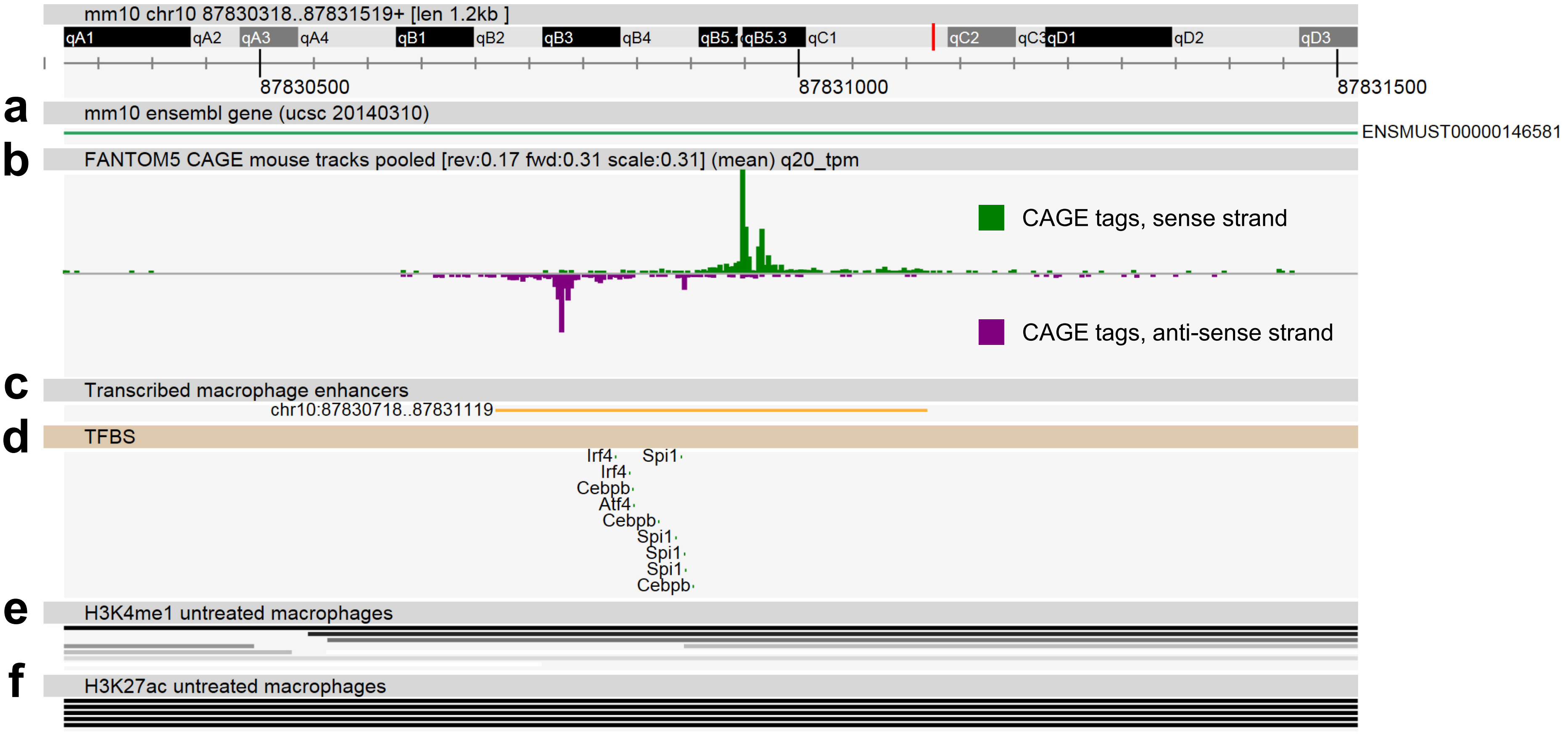

Figure S9. M(IL-4/IL-13) marker enhancer associated to Igf1 M(IL-4/IL-13) marker gene. ZENBU genome browser view shows the enhancer and 400bp flanking regions. The enhancer has macrophage-specific eRNA expression; see also Figure S8. **a** The enhancer is located in antisense RNA Igf1os. **b** CAGE signal in 184 macrophage samples, split by strand. **c** Enhancer region. **d** Summits of transcription factor binding sites; based on ChIP-seq data that were used for TFBS over-representation analysis, see Methods. **e, f** Significant ChIP-seq peaks for enhancer-specific chromatin marks that were used to define ChIP-seq-based enhancers, see Methods.

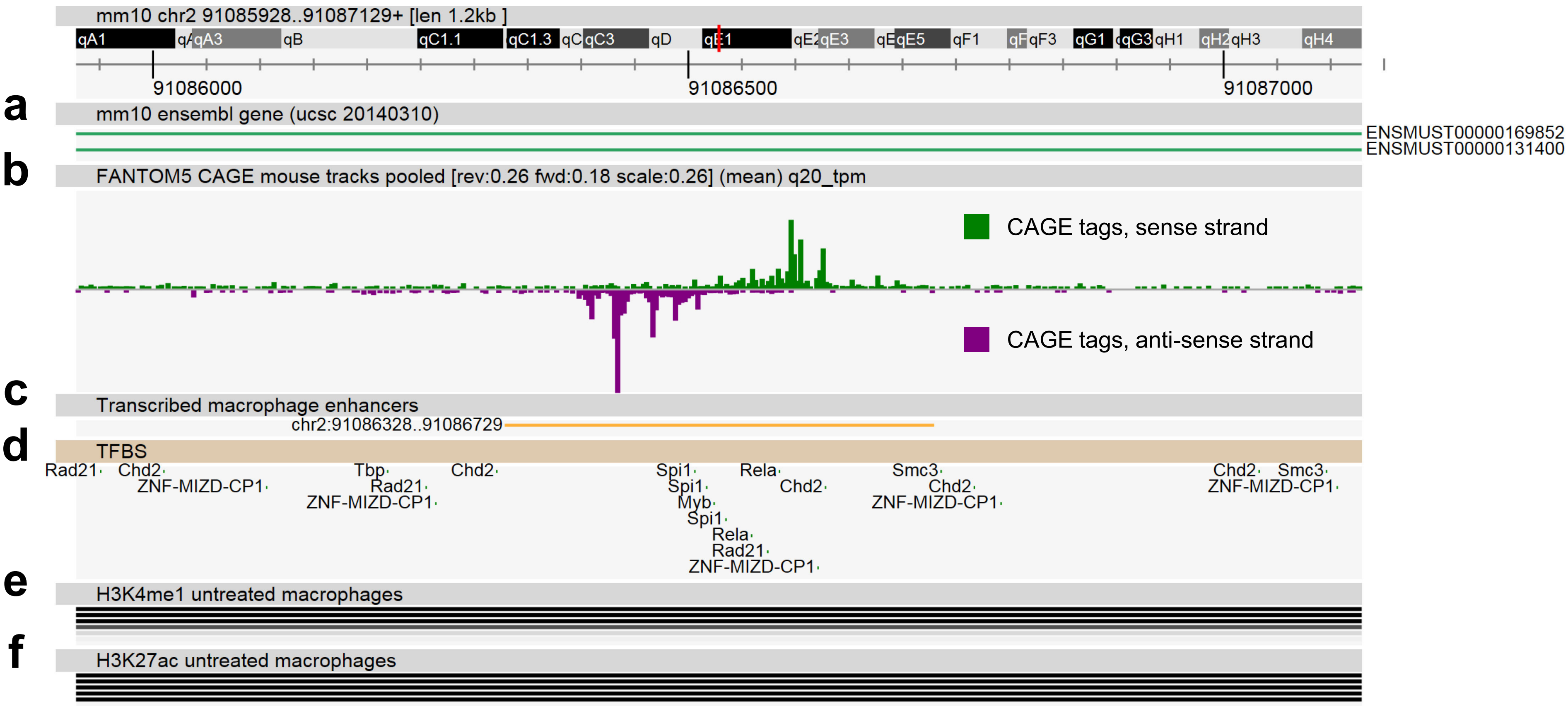

Figure S10. Macrophage-specific enhancer, associated to Spi1 gene. ZENBU genome browser view shows the enhancer and 400bp flanking regions. **a** The enhancer is located in an intron of its target gene Spi1. **b** CAGE signal in 184 macrophage samples, split by strand. **c** Enhancer region. **d** Summits of transcription factor binding sites; based on ChIP-seq data that were used for TFBS over-representation analysis, see Methods. **e, f** Significant ChIP-seq peaks for enhancer-specific chromatin marks that were used to define ChIP-seq-based enhancers, see Methods.
